# Metal-Based Nanoparticles for Cancer Metalloimmunotherapy

**DOI:** 10.3390/pharmaceutics15072003

**Published:** 2023-07-21

**Authors:** Ivan Hardianto Suliman, Kidong Kim, Weihsuan Chen, Yubin Kim, Jeong-Hyun Moon, Sejin Son, Jutaek Nam

**Affiliations:** 1 College of Pharmacy, Chonnam National University, Gwangju 61186, Republic of Korea; 2 Department of Biological Sciences, Inha University, Incheon 22212, Republic of Korea; 3 Department of Biological Sciences and Bioengineering, Industry-Academia Interactive R&E Center for Bioprocess Innovation, Inha University, Incheon 22212, Republic of Korea

**Keywords:** cancer, immunotherapy, metal ion, immune cell regulation, metal-based nanoparticle, metalloimmunotherapy

## Abstract

Although the promise of cancer immunotherapy has been partially fulfilled with the unprecedented clinical success of several immunotherapeutic interventions, some issues, such as limited response rate and immunotoxicity, still remain. Metalloimmunotherapy offers a new form of cancer immunotherapy that utilizes the inherent immunomodulatory features of metal ions to enhance anticancer immune responses. Their versatile functionalities for a multitude of direct and indirect anticancer activities together with their inherent biocompatibility suggest that metal ions can help overcome the current issues associated with cancer immunotherapy. However, metal ions exhibit poor drug-like properties due to their intrinsic physicochemical profiles that impede in vivo pharmacological performance, thus necessitating an effective pharmaceutical formulation strategy to improve their in vivo behavior. Metal-based nanoparticles provide a promising platform technology for reshaping metal ions into more drug-like formulations with nano-enabled engineering approaches. This review provides a general overview of cancer immunotherapy, the immune system and how it works against cancer cells, and the role of metal ions in the host response and immune modulation, as well as the impact of metal ions on the process via the regulation of immune cells. The preclinical studies that have demonstrated the potential of metal-based nanoparticles for cancer metalloimmunotherapy are presented for the representative nanoparticles constructed with manganese, zinc, iron, copper, calcium, and sodium ions. Lastly, the perspectives and future directions of metal-based nanoparticles are discussed, particularly with respect to their clinical applications.

## 1. Introduction

Cancer remains a major public health concern throughout the world. According to GLOBOCAN data, 19.3 million new cases and almost 10.0 million cancer-related deaths were recorded in 2020 [1]. Despite vast advances in the field, traditional clinical cancer therapy, such as chemotherapy, radiation therapy, and surgery, has shown limited therapeutic efficiency with considerable side effects due to a lack of specificity [2,3]. In recent years, cancer immunotherapy has emerged as a new clinical standard for many types of cancers [4], in which immune cells are trained to specifically locate cancer cells with systemic immune surveillance and generate anticancer immune responses for the elimination and long-term regression of local and metastatic tumors [5]. The therapeutic potential of cancer immunotherapy has been validated by the unprecedented bench-to-bedside clinical success of immune checkpoint inhibitors (ICIs), chimeric antigen receptor (CAR) T cell therapy, and oncolytic viruses against several tumors that have been refractory to other standard treatments [4,6]. However, cancer immunotherapy still faces challenges, such as toxicity and a low response rate [5]. For instance, most patients experience various symptoms of mild to severe immune-related adverse events, while only 20–30% benefit from ICIs [7]. Therefore, novel approaches for safe and effective cancer immunotherapy have been called for to provide more widespread clinical applications.

Metal ions are essential elements of host homeostasis and also play an important role in the regulation of the immune system [8]. They have inspired a new form of cancer immunotherapy called metalloimmunotherapy, which aims to harness the immunomodulatory features of metal ions for cancer immunotherapy [9]. However, metal ions are small charged species; thus, they exhibit physicochemical properties that present unfavorable in vivo pharmacological and drug-like activity. Therefore, effective metalloimmunotherapy requires the engineering of metal ions into suitable pharmaceutical forms that can improve the intrinsically poor in vivo performance of metal ions [9]. Nanomedicine offers promising tools to reshape metal ions into more drug-like formulations with nano-enabled engineering approaches, which can modulate the in vivo pharmacokinetic profiles of metal ions and target immune cells, reduce off-target toxicity, and improve therapeutic efficacy [10]. Accordingly, recent studies have demonstrated the potential of nanomedicine-based cancer metalloimmunotherapy. In this review, we illustrate the basic interaction between the immune system and cancer cells and the role of metal ions in the regulation of immune cells. We subsequently present recent preclinical studies that have developed and utilized metal-based nanoparticles for cancer metalloimmunotherapy and discuss the perspectives and future directions of this novel approach.

## 2. The Immune System and Cancer

The immune system is composed of innate and adaptive immunity that cooperate to recognize and defend the body against foreign/non-self-substances and infect-ed/malignant cells to maintain homeostasis [11,12]. The innate immune system is the first line of defense against infection, responding to and eliminating harmful immunogens in a rapid and nonspecific manner [13,14]. Short-lived innate immunity subsequently bridges the activation of the adaptive immune system for specific and durable immune responses. Adaptive immunity can provide robust long-term protection against repeated encounters with pre-exposed antigens by the immunological memory effect, which is a cardinal feature of immunotherapy for durable and effective disease control [13,15].

**Figure 1:**
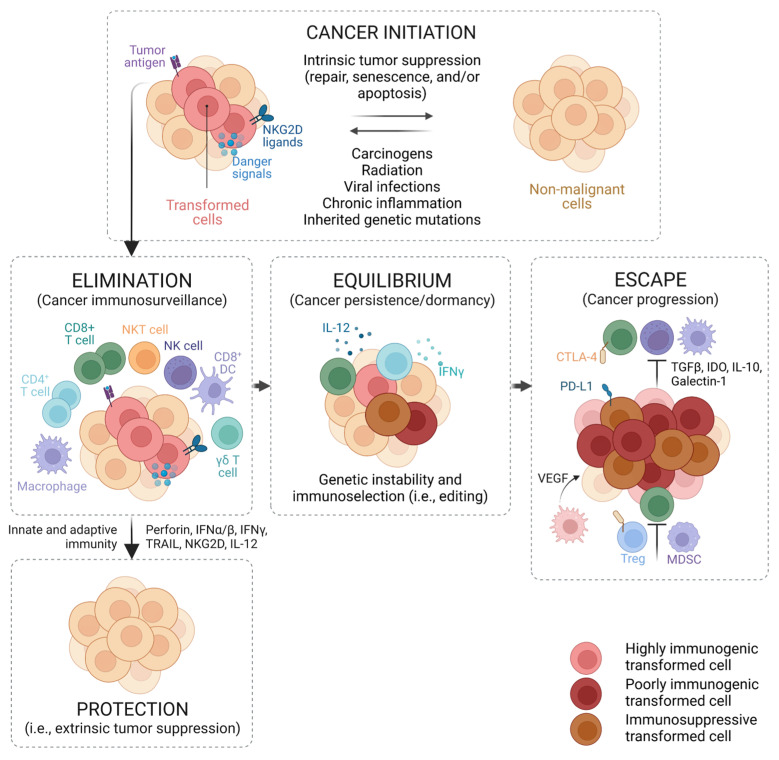
Cancer immunoediting. Adapted with permission from [16].

Since the concept of cancer immunosurveillance was first introduced more than a century ago [12], researchers have developed the immunoediting theory to explain tumor outgrowth under active immune functions in immunocompetent hosts (Figure 1) [16]. The host immune cells of innate and adaptive arms, including natural killer (NK) cells, dendritic cells (DCs), macrophages, and T cells, are constantly searching for malignant cancer cells in systemic circulation; this is a process called immunosurveillance, which can ultimately lead to the recognition and elimination of cancer cells via anticancer immune responses [12,17,18]. This initial phase associated with cancer immunosurveillance is called elimination, as it consists of eliminating cancer cells to provide protection against tumor growth. However, the heterogeneity and genetic instability of cancer cells often prevent complete tumor eradication; the constant immune pressure results in the evolution and selection of cancer cell subclones that can resist anticancer immune responses [16,19,20]. Such cancer cell variants may survive immunosurveillance and progress toward the equilibrium phase, where cancer cells remain functionally dormant or continue to evolve into new subclones harboring immune escape mutations such as a loss of antigen presentation and an increased expression of programmed death ligand-1 (PD-L1) [16,19,20]. These immune-edited tumors can then enter the escape phase, where the immune system is unable to control their growth, and the tumors become clinically apparent [20]. Some tumors may also escape immunosurveillance by recruiting immunosuppressive leukocytes such as regulatory T cells (Tregs) and myeloid-derived suppressor cells (MDSCs), which inhibit anticancer immune cells [21]. Cancer immunotherapy aims to increase the quality and quantity of anticancer immune cells to newly prime or reinforce existing anticancer immune responses to eliminate tumors in the equilibrium or escape phase. Some key immune cells for anticancer or suppressive immune responses are described below.

### 2.1. Antigen-Presenting Cells

DCs and macrophages not only play central roles in innate immune responses but they are also primary antigen-presenting cells (APCs) that induce antigen-specific T cell immunity by presenting cancer antigens in the context of major histocompatibility complex (MHC) molecules [22]. They require maturation signals such as pathogen-associated molecular patterns (PAMPs) and damage-associated molecular patterns (DAMPs) for immune activation [23]. These signals can be recognized by pattern recognition receptors (PRR), including Toll-like receptors (TLRs), retinoic acid-inducible gene I-like receptors (RLRs), NOD-like receptors (NLRs), and stimulator of interferon genes (STING) [22]. Upon maturation, DCs and macrophages exhibit an elevated expression of MHC molecules and costimulatory molecules (B7-1/B7-2) for the priming of T cells, and they secrete cytokines and chemokines that further activate T cells and enhance their migration to peripheral lymphoid organs or to the target site [24,25].

Among the major DC subsets, which include conventional DCs (cDCs), plasmacytoid DCs (pDCs), and monocyte-derived DCs (moDCs), cDCs are especially proficient at priming T cell responses [24,26]. cDCs can be subdivided into cDC1 and cDC2; cDC1s mainly induce cell immunity via CD8^+^ T cells, whereas cDC2s induce humoral immunity via CD4^+^ T cells [22,26]. cDC1s can also release interleukin (IL)-12 and promote interferon (IFN)-γ production and the activation of NK cells [26,27]. Furthermore, tumor-infiltrating cDC1s can produce chemokines such as the CXC chemokine ligand (CXCL) 9 and CXCL10 to attract CD8^+^ T cells into the tumor microenvironment (TME), representing an essential role of DCs for cancer immunotherapy [22]. Conversely, macrophages are highly plastic cells capable of polarizing into different functional subtypes, including the classically activated M1 and alternatively activated M2 subtypes [28]. M1 macrophages can be activated by IFN-γ, TNF-α, and maturation signals [29,30], and they secrete pro-inflammatory cytokines such as TNF-α, IL-1α, IL-1β, IL-6, IL-12, IL-18, and IL-23 [31]. M1 macrophages are known for their tumoricidal activity and their promotion of the activation of cytotoxic T cells. In contrast, M2 macrophages are activated by IL-4, IL-10, IL-13, and glucocorticoid hormones, and they facilitate immune suppression via the anti-inflammatory response [14]. Importantly, M2 macrophages constitute the major immune cell population in the TME; tumor-associated macrophages (TAMs) support the initiation, progression, and metastasis of tumors via the down-regulation of pro-inflammatory responses and by promoting angiogenesis and connective tissue remodeling via the release of various immunosuppressive cytokines, enzymes, and chemokines [7].

### 2.2. Effector Immune Cells

**Figure 2:**
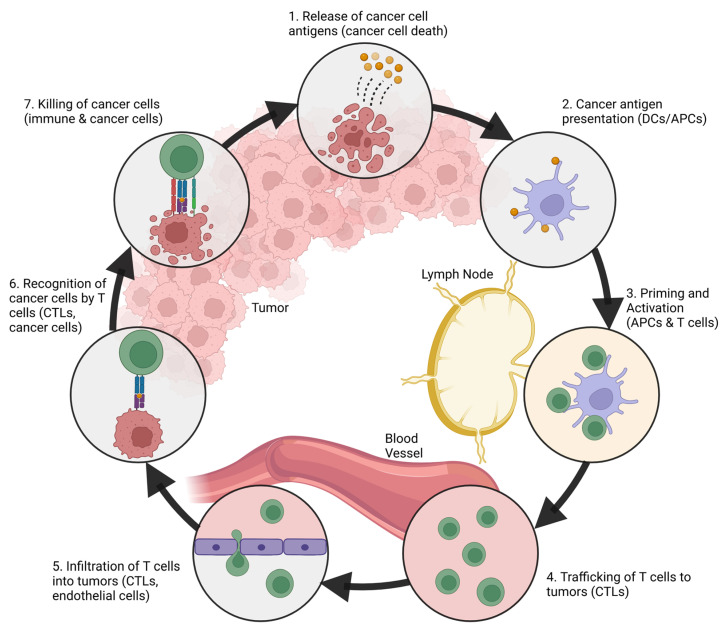
Cancer immunity cycle. Adapted with permission from [33].

CD8^+^ T cells or cytotoxic T lymphocytes (CTLs) are the key effector cells for the adaptive immune response, which can directly recognize and eliminate cancer cells in an antigen-specific manner [29,32]. The multistep immune cell–cancer cell and immune cell–immune cell interactions that are involved in the killing of cancer cells via CTL are referred to as the ‘cancer-immunity cycle’ (Figure 2) [33]. In the first step of the cycle, cancer cell antigens released by spontaneous cell death are captured by professional APCs such as DCs (possibly accompanied by the co-release and uptake of DAMP-based maturation signals). APCs then process antigen epitopes and present them to CD8^+^ T cells in the context of MHC I molecules in peripheral lymphoid organs [34]. Naïve T cells receive an antigenic signal via the T-cell receptor (TCR) and are primed and activated along with the co-stimulatory signals provided by APC maturation [18]. The activated T cells then migrate and infiltrate the tumor bed, recognize cancer cells via their cognate antigens bound to MHC I molecules, and exert cytotoxic activity for the induction of apoptosis [18,33]. As a consequence, cancer cells undergo immunogenic cell death, where dying cancer cells can release additional cancer cell antigens and DAMP-based maturation signals for the continued and sustained activation of the cycle [33].

NK cells can also directly engage and eliminate cancer cells [35,36]. Unlike CTLs, NK cells are innate immune cells and therefore do not require antigenic signals; their function is dependent on the repertoire and balance of activating and inhibitory receptor signaling [37]. For example, the inhibitory killer cell immunoglobulin-like receptor (KIR) can suppress NK cell activity after the recognition of MHC I molecules, which prevents the immune response and the subsequent collateral damage to healthy cells expressing MHC I [36,37]. Conversely, activating receptors can engage their ligands to promote NK cell activity. These include NK group 2 member D (NKG2D), such as MHC class I chain-related A and B (MICA/MICB) and several UL-16 binding proteins (ULBPs), natural cytotoxicity receptors (NCRs) like NKp30 and NKp46, and DNAX-activating molecule (DNAM1) [18,37,38]. Considering that cancer cells often down-regulate MHC I expression and up-regulate ligands for NK cell activating receptors during the immunoediting process, NK cells can effectively recognize cancer cells and subsequently undergo activation to generate robust anticancer immune responses in synergistic and/or complementary action with CTLs [18,36]. Furthermore, NK cells express Fc receptors that can bind to the common Fc region of immunoglobulins and induce antibody-dependent cellular cytotoxicity to eliminate immunoglobulin-targeted cells, thus allowing the elimination of cancer cells coated with cancer-specific antibodies produced de novo [35,38].

CTLs and NK cells can exert anticancer effector activity through the release of cytotoxic granules containing perforin and granzymes that are transmitted directly to cancer cells on contact [36]. They can also kill cancer cells via death receptor-induced cell apoptosis and the secretion of cytokines that inhibit tumor angiogenesis and further activate innate and adaptive immune responses [35,39,40].

### 2.3. Immunosuppressive Cells

Tumors in the escape phase often harbor various immunosuppressive cells that aid in immune evasion and tumor progression [41]. Among the best-characterized immunosuppressive cells are TAMs, MDSCs, and Tregs [30]. Consisting mainly of M2 macrophages, TAMs originate from monocytes that are recruited by tumor-derived cytokines, chemokines, and growth factors and then differentiate in response to IL-4, IL-10, and IL-13 [30]. They express CD68, CD163, CD204, and CD206 and produce a high level of IL-10 and arginase 1 (ARG-1), which are associated with a poor prognosis of solid tumors [42]. TAMs can negatively affect DCs and CTLs with cytokine regulation, express inhibitory ligands such as PD-L1 and B1-H4, and recruit Tregs by secreting chemokines [41]. MDSCs are immature myeloid cells that secrete various immunosuppressive substances, including ARG-1 and anti-inflammatory cytokines such as IL-10 and transforming growth factor-β (TGF-β), which upregulate PD-L1 expression and expand Tregs [10,30,43]. As a result, MDSCs disrupt T cell activation, DC antigen presentation, M1 macrophage polarization, and NK cell cytotoxicity [30]. The presence of MDSCs in the TME has been associated with drug resistance and metastatic progression, underscoring their pro-tumor effects [44,45,46]. Tregs are a subset of CD4^+^ T cells with critical immunosuppressive functions for maintaining normal immune homeostasis and self-tolerance [47]. They are attracted to hypoxic environments intrinsic to tumors, where they suppress anticancer immune cells; therefore, they are considered a significant barrier to cancer immunotherapy [48,49]. Tregs produce immunosuppressive cytokines such as IL-10, IL-35, and TGF-β; deplete IL-2; and express negative co-stimulatory molecules that suppress T cells [30,41]. Other immunosuppressive cells implicated in the tumor immune escape include N2 neutrophils and regulatory DCs (DCregs) [41]. N2 neutrophils suppress CD8^+^ T cells by releasing ARG-1, which facilitates the degradation of extracellular arginine essential for T cell function [41]. DCregs can induce T cells to undergo cell cycle arrest or apoptosis and promote Treg induction through the secretion of indoleamine-2,3-dioxygenase (IDO) [50]. Furthermore, they can also hinder T cell cytotoxicity through the production of TGF-β and IL-10 and the expression of PD-L1 and PD-L2 [51].

## 3. Metal Ions in the Host Response and Immune Modulation

Metal ions play an important role in the regulation of host homeostasis [52,53]. Some transition metal ions such as Fe^2+^, Mn^2+^, and Zn^2+^ are also essential elements in bacteria that serve as a cofactor to catalyze, stabilize, and activate many proteins and enzymes involved in vital physiological processes such as DNA replication/transcription and central metabolism [54]. Intriguingly, they can trigger host responses that are potentially associated with the inhibition of bacterial pathogens; the innate defense system not only actively exocytose these ions and thus sequester from intracellular pathogens but also accumulate them in the phagolysosomes to kill the bacteria, possibly by overdose toxicity or reactive oxygen species (ROS) production via a redox reaction [54,55]. Accordingly, studies have suggested that Cu ions accumulate in macrophage phagolysosomes via transporter proteins and induce oxidative damage to disrupt pathogens and clear infection [54].

The impact of metal ions on the activation of innate immunity has been demonstrated with inflammasome-mediated responses. The inflammasome is an oligomeric protein complex that activates caspase-1 for the maturation of IL-1β and IL-18β and subsequent pro-inflammatory responses [56]. In particular, inflammasomes that contain the nod-like receptor (NLR) family member NLRP3 can respond to several metal ions such as Ca^2+^, Na^+^, and K^+^. An increase in extracellular Ca^2+^ levels has been observed at sites of infection and ischemic necrosis to activate the NLRP3 inflammasome, suggesting that Ca^2+^ is a DAMP signal released by damaged cells [57]. Na^+^ and K^+^ can also trigger the formation of the NLRP3 inflammasome by inducing an ionic imbalance within cells [56,58]. Intracellular K^+^ depletion has been shown to be necessary and sufficient to activate NLRP3 inflammasomes, which can be caused by active K^+^ efflux and the formation of membrane pores permeable to K^+^ [56]. Conversely, Na^+^ influx can dysregulate the Na^+^/K^+^ gradient and promote K^+^ efflux, thus leading to the activation of the NLRP3 inflammasome [58]. In addition, metal ions can also directly stimulate or potentiate the cyclic GMP-AMP synthase (cGAS)-STING pathway. As a critical innate defense mechanism against viral infection, cGAS can detect cytosolic dsDNA, a hallmark of the virus, and subsequently catalyze the synthesis of cyclic GMP-AMP (cGAMP), which then binds to STING and triggers the downstream signaling pathway, stimulating the pro-inflammatory responses and production of type I IFNs [59]. Mn^2+^ has been shown to enhance sensitivity to DNA and the enzymatic activity of cGAS while also promoting the binding of cGAMP to STING, thus potentiating multiple factors in the cGAS-STING pathway [60]. Zn^2+^ has also been shown to increase cGAS activity by facilitating the phase transition of cGAS and stabilizing the cGAS-dsDNA complex [59]. 

Metal ions can also regulate the pivotal immune cells in the adaptive immune system. Zn^2+^ is essential for the development and function of T cells and B cells, where its deficiency causes thymic atrophy and subsequent T cell lymphopenia, disrupts T cell subtype differentiation potentially causing immune dysfunction, impairs T cell activation and expansion, and reduces B cell maturation and antibody production [55]. In addition, Zn^2+^ exhibits various immunological functions related to the priming of adaptive immunity, including polymorphonuclear cell recruitment and phagocytic activation, the maturation and differentiation of DCs and NK cells, and pro-inflammatory cytokine production [55]. For the activity of CTLs, Mg^2+^ promotes the up-regulation of lymphocyte function-associated antigen 1 (LFA-1) and costimulatory T cell receptors to enhance their cytotoxicity [4]. TCR signaling is a key step in initiating adaptive immune responses by T cells, whereby Ca^2+^ plays a critical role by manipulating phospholipids and signaling proteins [61]. Specifically, Ca^2+^ can neutralize anionic phospholipids and subsequently increase the accessibility of immunoreceptor tyrosine-based activation motif (ITAM), leading to enhanced TCR sensitivity to antigenic signals by the amplification of downstream signaling [61]. Additionally, T cells in the immune synapse promote the influx of Ca^2+^ to further regulate the phosphorylation of cytoplasmic signaling proteins and amplify signaling cascades associated with the activation and effector function of T cells [61]. In contrast, K^+^ serves as an ionic checkpoint that attenuates T cell activity; elevated intracellular K^+^ concentration inhibits the activation of effector T cells while enriching Tregs [59]. In vitro studies with human cells and in vivo studies with tumor-bearing mice have shown that the depletion of excessive K^+^ can restore T cell function for anticancer immune responses [59]. Conversely, high levels of K^+^ induce a state of caloric restriction by limiting the uptake and metabolism of nutrients, which triggers a starvation response that promotes metabolic and genetic reprogramming toward T cell memory formation and stemness [62]. The stemness of T cells is characterized by their ability to persist, self-renew, clonally repopulate, and exhibit pluripotency for their progeny to acquire effector functions, which is beneficial for the tumoricidal capacity of T cells [62]. This suggests that K^+^ has a dual effect on T cell regulation, which should be appropriately balanced for effective cancer immunotherapy.

## 4. Metal-Based Nanoparticles for Cancer Metalloimmunotherapy

Nanotechnology has established a variety of nanoparticle platforms that can positively modulate the in vivo pharmacological profile of drug molecules with nano-enabled engineering approaches [63,64]. Metal-based nanoparticles have also been developed and applied to cancer imaging and therapy based on their intrinsic electromagnetic properties and direct cytotoxic activity in cancer cells [3]. Recently, the emergence of metalloimmunotherapy has spurred renewed interest in the application of metal-based nanoparticles for cancer immunotherapy with intrinsic or extrinsic anticancer and immunomodulatory features of metal ions. Below, we introduce representative preclinical studies that have utilized metal-based nanoparticles for cancer metalloimmunotherapy (Table 1). We categorized metal-based nanoparticles by their principal cellular and molecular mechanisms associated with the induction of antitumor immune responses.

### 4.1. Manganese- and Zinc-Based Nanoparticles for cGAS-STING Activation

**Figure 3:**
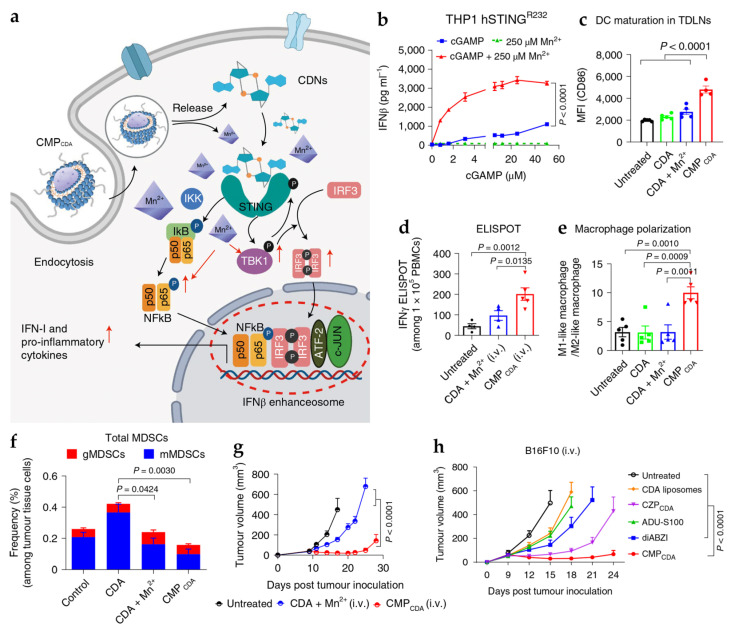
Antitumor activity of CMP_CDA_. (**a**) Schematic diagram of the activation of cGAS-STING pathway by CMP_CDA_. (**b**) STING activation in THP1 cells expressing hSTING^R232^. (**c**–**f**) The levels of immune cells and (**g**) antitumor efficacy of CMP_CDA_ in CT26 tumor-bearing mice. (**h**) Antitumor efficacy of CMP_CDA_ in B16F10 tumor-bearing mice. Reprinted with permission from [9].

Manganese-containing nanoparticles can stimulate innate and adaptive immunity through the cGAS-STING pathway [65,66]. Sun et al. showed that Mn^2+^ can potentiate the activity of the cyclic dinucleotide (CDN)-based STING agonist by an order of magnitude in multiple human STING haplotypes (Figure 3) [9]. The authors designed the self-assembled CDN-Mn^2+^ nanoparticle (CMP) to take advantage of a nanoparticle formulation to improve CDN and Mn^2+^ metabolic stability, cell permeability, intracellular activity, and in vivo performance. CMP was demonstrated to trigger anticancer immune responses through NF-kB and IRF3, the downstream signaling of the cGAS-STING pathway. It induced DC maturation, cytokine production, and M1 macrophage polarization, leading to the activation of CD8^+^ T cells and inhibition of immunosuppressive MDSCs for remarkable antitumor efficacy in multiple difficult-to-treat murine tumor models. Liu et al. developed biomineralized manganese oxide nanoparticles (Bio-MnO_2_ NPs) in combination with radiotherapy (RT), showing that it could induce apoptosis and the DNA release of cancer cells to activate the cGAS-STING pathway [67]. Bio-MnO_2_ NPs not only sensitized RT to stimulate cGAS-STING signaling but also relieved tumor hypoxia and generated ROS with the release of Mn^2+^ and oxidation of hydrogen peroxide into oxygen. As a result, RT plus Bio-MnO_2_ NPs elicited robust anticancer immunity while alleviating the immunosuppressive TME, significantly inhibiting tumor growth in a murine non-small-cell lung cancer (NSCLC) model. Similarly, Hou et al. constructed doxorubicin (DOX)-loaded, phospholipid (PL)-coated amorphous manganese phosphate nanoparticles for synergistic immune stimulation; DOX could trigger DNA damage that activated cGAS-STING signaling, which, in turn, was potentiated by Mn^2+^ [68]. The hybrid nanoparticles effectively inhibited primary growth, relapse, and the distant metastasis of murine 4T1 breast tumors with strong local and systemic immune responses, leading to improved survival among tumor-bearing mice [68].

**Figure 4:**
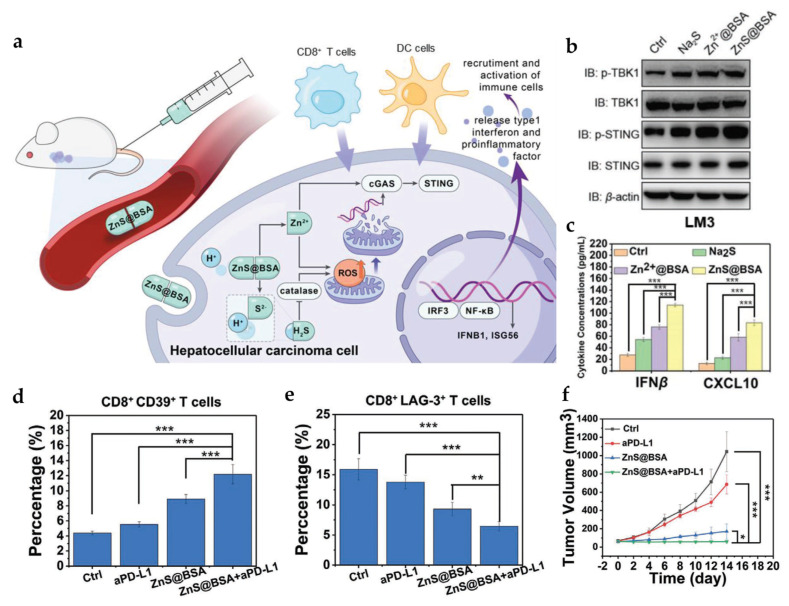
Antitumor activity of ZnS@BSA. (**a**) Schematic diagram of ZnS@BSA function in a HCC cell. (**b**) Western blotting analysis of STING marker expression in LM3 cells. (**c**–**e**) The levels of cytokines and immune cells in randomly selected tumor samples and (**f**) antitumor efficacy in HCC tumor-bearing mice. Reprinted with permission from [69].

Zinc ions can also directly and indirectly stimulate cGAS-STING activation and anticancer immune responses [69]. Wang et al. showed that zinc oxide nanoparticles (ZnO NPs) could promote cancer cell apoptosis with the production of ROS [70]. ZnO NPs enhanced cellular uptake and the tumor spheroid penetration of DOX by inhibiting P-glycoprotein (Pgp)-mediated drug efflux and three-dimensional (3D) spheroid architecture-induced drug resistance, leading to the efficient killing of cancer and cancer stem cells. In addition, ZnO NPs activated macrophages and boosted anticancer immunity while decreasing the toxicity of DOX against macrophages. Cen et al. synthesized pH-responsive ZnS@BSA (bovine serum albumin) nanoclusters using a diffusive self-assembly approach that can release zinc and sulfur ions in an acidic TME (Figure 4) [69]. The released Zn^2+^ activated cGAS/STING signaling, whereas S^2−^ formed H_2_S gas that inhibited catalase and cooperated with zinc ions to generate ROS in hepatocellular carcinoma (HCC) cells. The intravenous administration of ZnS@BSA significantly inhibited HCC tumor growth through anticancer immune responses that could be potentiated by the PD-L1 antibody. It also conferred long-term protection against tumor recurrence, indicating the induction of robust and durable anticancer immunity. Another study by Zhang et al. constructed Zn^2+^ into a nano-formulation using a layered double hydroxide (LDH) that could neutralize acidity in tumor tissues and induce cell apoptosis [71]. Zn^2+^ doping in LDH promoted ROS production to accelerate mitochondrial damage and immunogenic cell death, leading to the activation of cGAS-STING and down-regulation of ‘don’t eat me’ signals (i.e., PD-L1 and CD47). As a result, Zn-LDH stimulated anticancer immune cells (M1-TAM, CTL, and NK cells) and efficiently inhibited tumor growth and the metastasis of murine models of melanoma and breast cancer [71].

### 4.2. Iron- and Copper-Based Nanoparticles for M1 Macrophage Polarization

**Figure 5:**
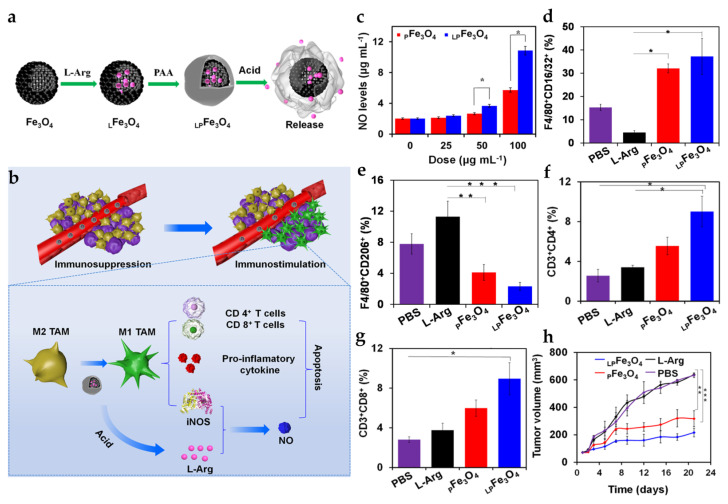
Antitumor activity of _LP_Fe_3_O_4_ NPs. Schematic diagram of (**a**) L-Arg loading and pH-responsive release and (**b**) M2-M1 macrophage polarization and NO production by _LP_Fe_3_O_4_ NPs. (**c**) NO levels of M2 macrophages treated with different formulations. (**d**,**e**) TAM phenotype (CD16/32 for M1 and CD206 for M2, respectively), (**f**,**g**) CD4^+^ and CD8^+^ T cells, and (**h**) antitumor efficacy in 4T1 tumor-bearing mice. Reprinted with permission from [78]. Copyright 2021 American Chemical Society.

Iron-based nanoparticles are mainly oxidation products that include magnetite (Fe_3_O_4_), maghemite (γ-Fe_2_O_3_), and mixed ferrites (MFe_2_O_4_ where M = Co, Mn, Ni, or Zn) [72]. Such iron oxide nanoparticles have proved effective for drug delivery and cancer theranostics due to their biological and electromagnetic properties [73,74,75]. Recently, it has also been demonstrated that iron oxide nanoparticles can elicit anticancer immune responses. Ferumoxytol, an FDA-approved iron oxide nanoparticle for the treatment of anemia, has been shown to promote the polarization of macrophages into the M1 phenotype [76]. The polarization of M1 is characterized by an increase in pro-M1 genes (TNFα, CD86), a decrease in pro-M2 genes (IL10, CD206) in vitro, and an up-regulation of CD80 expression in vivo by macrophages. Furthermore, ferumoxytol could catalyze ROS generation through the Fenton reaction with hydrogen peroxide secreted by M1 macrophages, leading to the apoptosis of cancer cells accompanied by the expression of caspase 3 [77]. In vivo mice models have demonstrated that ferumoxytol suppresses the tumor growth of early mammary cancers and inhibits the liver and lung metastasis of small-cell lung cancer. In another study, Wu et al. used hollow Fe_3_O_4_ nanoparticles for the loading of _L_-arginine (_L_-Arg), which was followed by poly(acrylic acid) (PAA) sealing to provide pH-responsive release in the acidic TME (Figure 5) [78]. M1 macrophages can efficiently convert _L_-Arg into NO with elevated levels and catalytic activity of inducible nitric oxide synthase (iNOS), allowing for NO-based gas therapy. Therefore, _L_-Arg could synergistically enhance the anticancer efficacy of iron oxide nanoparticles by utilizing their M2-to-M1 reprogramming capability. Chen et al. developed iron oxide nanoparticles to boost cancer vaccine activity [75]. They synthesized iron oxide-mesoporous organosilica core–shell nanospheres and then loaded protein antigen in the large-pore organosilica shell layer for vaccine application. The study showed that the hybrid nanoparticles could induce M2-to-M1 macrophage polarization and antigen-specific T cell immunity, leading to strong anticancer immune responses and the complete prevention of tumor development. Recent studies have also demonstrated that copper ions can induce M1 macrophage polarization [79,80]. Xu et al. showed that copper sulfide (CuS) nanoparticles can trigger ROS generation via mitochondrial fission and the Fenton reaction, leading to the activation and M1 polarization of bone marrow-derived macrophages (BMDMs) through the classical IKK-dependent NF-κB pathway [81]. The adoptive transfer of the ex vivo CuS nanoparticle-stimulated BMDMs prolonged the median survival time of melanoma-bearing mice, enhancing the phagocytic and digestive ability of BMDMs to the cancer cells. Another study by Ge et al. produced CuS nanoparticles using ovalbumin proteins (OVA) as a template [82]. The authors demonstrated that CuS@OVA nanoparticles can not only promote M1 macrophage polarization in the acidic TME with the release of copper ions but also induce DC activation and maturation for the priming of T cells. Furthermore, CuS@OVA allowed for mild-temperature photothermal therapy, which, together with the administration of the anti-programmed death-1 antibody (aPD-1), efficiently suppressed the primary growth and distant metastasis of B16-OVA melanoma in mice.

### 4.3. Calcium- and Sodium-Based Nanoparticles for Ion Overloading Effect

**Figure 6:**
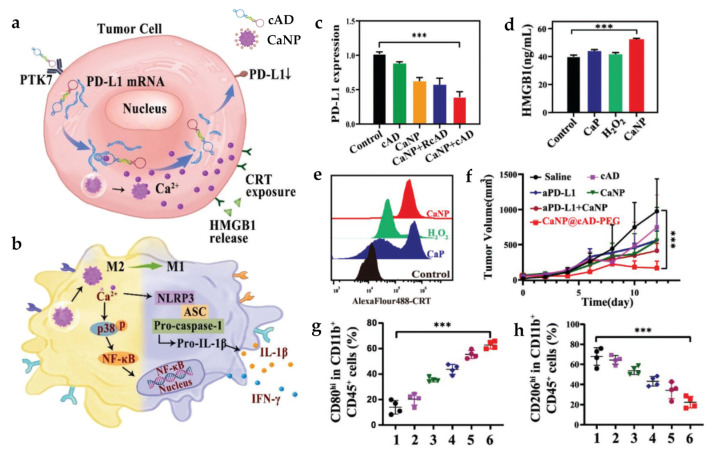
Antitumor activity of CaNP@cAD-PEG. Schematic diagram of (**a**) CaNP function in a tumor cell and (**b**) TAM reprogramming via Ca^2+^ interference. The expression of (**c**) PD-L1 and (**d**,**e**) DAMPs (CRT and HMGB1) in B16 cells. (**f**) Antitumor efficacy in B16 tumor-bearing mice. The levels of (**g**) M1 and (**h**) M2 macrophages. Reprinted with permission from [85].

Calcium-based nanoparticles can exhibit various anticancer and immunomodulatory effects caused by Ca^2+^ overloading. An et al. developed honeycomb OVA@CaCO_3_ nanoparticles (HOCNs) that can be decomposed in an acidic TME and exert several functionalities associated with the release of Ca^2+^; they alleviate tumor acidity, support autophagy, and promote DAMP secretion from cancer cells [83]. When employed with the chemotherapeutic drug mitoxantrone, HOCN induced the maturation and antigen presentation of DCs and subsequently activated CTLs and effector memory T cells, leading to robust antitumor immunity for the regression of both primary and distant tumors. Recently, Zheng et al. reported curcumin-loaded CaCO_3_ nanoparticles for pyroptosis-based cancer immunotherapy [84]. Curcumin could stimulate the release of Ca^2+^ from the endoplasmic reticulum into the cytoplasm, which could later accumulate and overload in the mitochondria. The surges of Ca^2+^ could break the dynamic Ca^2+^ equilibrium in the mitochondria, leading to ROS induction, cytochrome C release, caspase 3 activation, gasdermin E cleavage, and eventually cell blebbing and pyroptosis. Ca^2+^ overloading-mediated pyroptosis elicited robust anticancer immune responses (marked by DC maturation and T cell activation), thereby efficiently inhibiting tumor growth. Calcium-based nanoparticles can also re-educate TAM via “Ca^2+^ interference”, allowing for M2-to-M1 phenotype switching. An et al. prepared and functionalized calcium peroxide nanoparticles (CaNPs) with a circular aptamer-DNAzyme conjugate (cAD) and a PEG shell (Figure 6) [85]. “Ca^2+^ interference” by CaNPs was found to trigger p38 phosphorylation, NF-κB p65 nuclear translocation, and NLRP3-inflammasome activation, resulting in M2-to-M1 polarization and adaptive immune responses. Furthermore, CaNP induced immunogenic cancer cell death and the release of DAMPs, and cAD mimicked endonuclease activity to block the translation of PD-L1 mRNA using Ca^2+^ as a cofactor. Together, the composite nanoparticles effectively inhibited primary tumor growth, prevented lung metastasis, and developed long-term immunological memory against tumor rechallenge.

**Figure 7:**
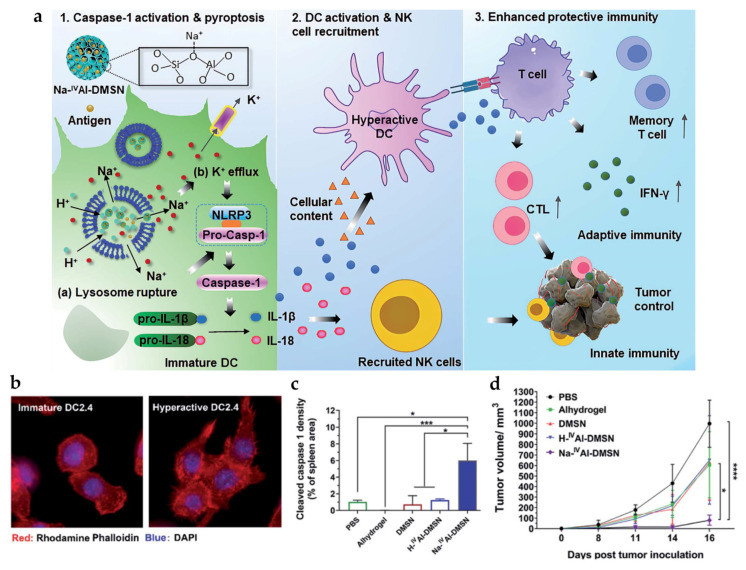
Antitumor activity of Na-^IV^Al-DMSN. (**a**) Schematic diagram of Na-^IV^Al-DMSN function in a DC. (**b**) Morphological changes in DC2.4 cells after hyperactivation. (**c**) Caspase-1 expression in the spleen of CT26 tumor-bearing mice. (**d**) In vivo tumor growth in a prophylactic tumor model. Reprinted with permission from [86].

A recent study by Tang et al. described the preparation of sodium-stabilized dendritic mesoporous aluminosilicate nanoparticles (Na-^IV^Al-DMSN) as DC pyroptosis modulators and antigen carriers for cancer vaccine applications (Figure 7) [86]. Once internalized by DCs, Na-^IV^Al-DMSN promoted H^+^/Na^+^ exchange in acidic lysosomes in a pH-responsive manner, leading to lysosomal rupture and subsequent Na^+^ influx/K^+^ efflux in cells. The intracellular ion perturbation induced caspase-1-dependent DC pyroptosis and inflammasome activation, which triggers the hyperactivation of bystander DCs, a superior state in priming T cells. Consequently, CT26 cancer cell lysate-loaded Na-^IV^Al-DMSN boosted immune responses in vivo (marked by the high level of activated DCs), cytotoxic NK cells, CTLs, and effector memory T cells, showing strong anticancer efficacy when administered in a prophylactic or therapeutic setting.

**Table 1:** Summary of metal-based nanoparticles and their preclinical applications for cancer metalloimmunotherapy.

Metal	Nanoparticle Formulation	Surface Modification and Drug Loading	Nanoparticle Characteristics	Target Cells	Route and Dose (In Vivo)	Properties and Outcomes	References
Mn	CMP_CDA_	SM: PEG-lipid bilayer	PS: 118 ± 41 nm PDI: 0.107 ZP: −2.49 ± 6.42 mV	In vitro DC: BMDCs In vitro tumor cells: THP1 In vivo tumor model: CT26, B16F10, NOOC1 mice	i.t.: CDA 5 µg, Mn^2+^ 2.5 µg i.v.: CDA 20 µg, Mn^2+^ 10 µg	Deliver STING agonist to immune cells and enhances its activity through the activation of the cGAS-STING pathway	[9]
	Bio-MnO_2_ NPs + RT	-	PS: 101 nm PDI: 0.122	In vitro tumor cells: A549, PC9, H520 In vivo tumor model: LLC mice	i.t.: 2 mg/kg + IR: 8 Gy	Relieve tumor hypoxia, activate the cGAS-STING pathway, and sensitize RT	[67]
	PL/APMP-DOX NPs	SM: PL layer DL: DOX	PS: ~180 nm PDI: 0.232 ZP: −23.8 ± 0.6 mV Pore Size: 3.88 nm	In vivo tumor model: 4T1 mice	i.v.: APMP 2.50 mg/kg, DOX 2.50 mg/kg	Activate the cGAS-STING pathway and carry DOX to induce DNA damage	[68]
Zn	ZnO/DOX	DL: DOX	In DMEM/10%FBS medium: PS: 62.02 ± 0.98 nm PDI: 0.31 ± 0.03 ZP: −16.06 ± 0.09 mV	In vitro tumor cells: DOX-sensitive (MDA-MB-231 and HeLa), DOX-resistant (NCI/ADR-RES and MES-SA/Dx5) In vitro macrophage: RAW264.7	-	Generate ROS and activate caspase 3/7, polarize macrophages into M1 phenotype, downregulate the CD44 expression of stem-like cancer cells, and carry DOX to kill cancer cells synergistically	[70]
	ZnS@BSA	-	PS ≈ 100 nm	In vitro tumor cells: HCC (LM3 and Hepa1-6) In vivo tumor model: Hepa1-6 mice	i.v.	Cooperate with H_2_S to accumulate ROS and activate the cGAS-STING pathway	[69]
	Zn-LDH	-	-	In vitro tumor cells: B16F10 and 4T1 In vitro DC: DC2.4 In vitro macrophage: RAW264.7 In vivo tumor model: B16F10 and 4T1 mice	PT: 1 mg	Activate the cGAS-STING pathway, block the autophagy pathway, and neutralize TME acidity	[71]
Fe	Ferumoxytol (Feraheme)	-	PS 30 nm	In vitro tumor cells: MMTV-PyMT In vitro macrophage: RAW264.7/BMDMs In vivo tumor model: MMTV-PyMT mice In vivo metastatic model: KP1 mice	MFP injection: 100 µL at a conc. of 2.73 mg Fe/mL i.v.: 10 mg Fe/kg	Polarize macrophages into M1 phenotype	[76]
	Positively and Negatively Charged SPIONs	-	For S+: PS: 19.4 ± 0.8 nm ZP: +44.72 mV For S-: PS: 21.3 ± 1.6 nm ZP: −27.31 mV	In vitro tumor cells: HT1080 In vitro macrophage: RAW264.7 In vivo tumor model: HT1080 mice	i.t.	Polarize macrophages into M1 phenotype	[87]
	_LP_Fe_3_O_4_ NPs	SM: PAA layer DL: _L_-Arg	PS: 229.4 ± 5.0 nm ZP: −31.1 ± 0.6 mV Pore Size: 3.5 nm	In vitro tumor cells: 4T1 In vitro macrophage: RAW264.7/BMDMs In vivo tumor model: 4T1 mice	i.v.: 20 mg Fe_3_O_4_/kg	Polarize macrophages into M1 phenotype and carry _L_-Arg to induce NO production	[78]
	IO-LPMONs-OVA vaccine + IO-LPMONs	-	PS: 360 nm Pore Size: 6.3 nm	In vitro tumor cells: SCC7 In vitro macrophage: RAW264.7 In vivo tumor model: EG7-OVA mice	s.c.: IO-LPMONs-OVA vaccine (100 µL, conc. 1 mg/mL) + i.t.: IO-LPMONs (100 µL, conc. 0.2 mg/mL)	Polarize macrophages into M1 phenotype and carry OVA for vaccination	[75]
Cu	CuS NPs	SM: PEG	PS ≈ 17 nm	In vivo tumor model: B16F10 mice	i.t.	Polarize BMDMs ex vivo into M1 phenotype for adoptive macrophage therapy	[81]
Cu	CuS@OVA	DL: OVA	PS: 16.3 ± 0.2 nm	In vitro tumor cells: B16-OVA In vitro DC: BMDCs In vitro macrophage: BMDMs In vivo tumor model: B16-OVA and orthotopic uveal B16-Luc mice	i.t.: CuS-OVA (10 or 2 µL, conc. 1 mg/mL) + i.p.: aPD-1 10 mg/kg	Polarize macrophages into M1 phenotype and activate DCs, serve as a photosensitizer for PTT, and augment the antitumor efficacy of aPD-1	[82]
Ca	MTX + HOCN	-	PS: 250 nm ZP: −7.6 mV	In vitro tumor cells: CT26 In vitro DC: DC2.4 In vivo tumor model: CT26 mice	i.v.: 40 mg/kg	Overcome the multiple barriers of DCs by disrupting autophagy inhibition, attenuating TME acidity, and releasing DAMPs	[83]
	CaNMs	DL: CUR	-	In vitro tumor cells: 4T1 In vivo tumor model: 4T1 mice	i.t.	Induce Ca^2+^ overloading-mediated pyroptosis to stimulate antitumor immune responses	[84]
	CaNP@cAD-PEG	SM: DSPE-PEG2000 DL: cAD	PS: 180 nm	In vitro tumor cells: B16 In vitro macrophage: BMDMs In vivo tumor model: B16 and CT26 mice	i.v.	Induce TAM re-education through multiple inflammation-related signaling pathways as well as NLRP3-inflammasome, promote cancer antigen release, and suppress PD-L1 expression	[85]
Na	Na-^IV^Al-DMSN	-	PS: ~240 nm	In vitro DC: DC2.4 In vivo tumor model: CT26 mice	s.c.	Induce intracellular ion perturbation for DC pyroptosis and hyperactivation, provoking enhanced NK cell-mediated innate immunity and both cellular and humoral adaptive immune responses	[86]

4T1, murine mammary carcinoma cell; A549, human lung adenocarcinoma cell; aPD-1, anti-programmed cell death-1 antibody; B16F10, murine melanoma cell; B16-Luc, murine melanoma cell labelled with luciferase; B16-OVA, murine melanoma cell expressing OVA; Bio-MnO_2_ NP, biomineralized MnO_2_ nanoparticle; BMDC, bone-marrow-derived dendritic cell; BMDM, bone-marrow-derived macrophage; cAD, circular aptamer-DNAzyme conjugate; CaNM, Ca^2+^ nanomodulator; CaNP@cAD-PEG, cAD-loaded DSPE-PEG2000-coated CaO_2_ nanoparticle; CDA, cyclic di-AMP; cGAS, cyclic GMP-AMP synthase; CMP_CDA_, CDA-Mn^2+^ particle; CT26, murine colorectal carcinoma cell; CUR, curcumin; DAMP, damage-associated molecular pattern; DC, dendritic cell; DL, drug loading; DMEM/10%FBS, Dulbecco’s modified Eagle’s medium complete medium containing 10% fetal bovine serum; DNA, deoxyribonucleic acid; DOX, doxorubicin; DSPE-PEG2000, 1,2-distearoyl-sn-glycero-3-phosphoethanolamine-N-PEG-2000; EG7-OVA, murine thymic lymphoma cell transfected with OVA DNA; H520, human lung squamous carcinoma cell; HCC, hepatocellular carcinoma; HeLa, human cervical cancer cell; HOCN, honeycomb OVA@CaCO_3_ nanoparticle; HT1080, human fibrosarcoma cell; IO-LPMON, Fe_3_O_4_-embedded large-pore mesoporous organosilica nanosphere; i.p., intraperitoneal; IR, irradiation; i.t., intratumoral; i.v., intravenous; KP1, small-cell lung cancer cell; _L_-Arg, L-Arginine; LLC, Lewis lung carcinoma cell; LM3, murine mammary adenocarcinoma cell; _LP_Fe_3_O_4_ NP, _L_-Arg-loaded PAA-coated hollow Fe_3_O_4_ nanoparticle; MDA-MB-231, human triple-negative breast adenocarcinoma cell; MES-SA/Dx5: multi-drug-resistant human uterine sarcoma cell; MFP, mammary fat pad; PTT, photothermal therapy; MMTV-PyMT, mouse mammary tumor virus-polyoma middle T antigen cell; MTX, mitoxantrone; Na-^IV^Al-DMSN, sodium-stabilized dendritic mesoporous aluminosilicate nanoparticle; NCI/ADR-RES, multi-drug-resistant human ovarian cancer cell; NLRP3, nod-like receptor family pyrin domain containing 3; NO, nitric oxide; NOOC1, immune checkpoint blockade-resistant murine tobacco-associated tumor cell; OVA, ovalbumin; PAA, poly(acrylic acid); PC9, human lung adenocarcinoma cell; PDI, poly-dispersity index; PD-L1, programmed death-ligand 1; PEG, poly(ethylene glycol); PL, phospholipid; PL/APMP-DOX NP, DOX-loaded PL-coated amorphous porous manganese phosphate nanoparticle; PS, particle size; PT, peritumoral; ROS, reactive oxygen species; RT, radiotherapy; s.c., subcutaneous; SCC7, murine squamous cell carcinoma cell; SM, surface modification; SPION, super-paramagnetic Fe_3_O_4_ nanoparticle; STING, stimulator of interferon genes; TAM, tumor-associated macrophage; THP1, human leukemia monocytic cell; TME, tumor microenvironment; Zn-LDH, Zn^2+^-doped layered double hydroxide nanoparticle; ZnO/DOX, DOX-loaded ZnO nanoparticle; ZnS@BSA, ZnS@bovine serum albumin nanocluster; ZP, zeta potential.

## 5. Conclusions

Metalloimmunotherapy offers a promising novel cancer treatment that involves utilizing metal ions to increase anticancer immune responses and anticancer efficacy. Metal-based nanoparticles can improve the pharmacological and drug-like properties of metal ions along with versatile nanoparticle engineering for the desired in vivo activity and functionality. The preclinical studies summarized in this review have demonstrated early promise, calling for further research on the clinical impact of these metal-based nanoparticles. The clinical development of metal-based nanoparticles should also consider cancer immunotherapy barriers and nanomedicine design principles to fulfil the full therapeutic potential of metalloimmunotherapy. In addition, combination therapy could be a promising approach to increse the utility of metal-based nanoparticles and improve their therapeutic outcomes with the rational selection of the combination counterparts.

## 6. Perspectives and Future Directions

Immunotherapy has proven to be effective in a range of cancers, including those refractory to other standard treatments. However, issues that significantly hamper clinical efficacy and ultimately lead to low response rates have emerged [88]. In particular, the TME poses a significant barrier for cancer immunotherapy. It harbors not only cancer cells but also immune cells, fibroblasts, and the extracellular matrix (ECM) in a compact organized structure, elements that are programmed by cancer cells to support tumor growth, metastasis, and migration [1]. The dense ECM layers in the periphery, together with high interstitial pressure, exclude anticancer immune cells from the tumor core (where cancer cells reside), thus preventing them from exerting effector function [4]. In addition, the TME operates abnormal biological cues that result in hypoxia, increased acidity, limited nutrient availability, and dysregulated ion concentrations, which induce the dysfunction and tolerance of anticancer immune cells while promoting immunosuppressive cells [2,3]. As a result, the TME impairs the activation, proliferation, and anticancer responses of effector cells and other anticancer immune cells crucial for cancer immunotherapy. Furthermore, cancer cells can evade anticancer immune responses through a variety of mechanisms, such as the expression of inhibitory receptors and ligands, loss of MHC and antigens, and secretion of suppressive cytokines and metabolites in the TME [2]. As described in this review, certain metal ions have the potential to stimulate innate and adaptive immune cells for robust anticancer immune responses [1]. In addition, they can directly kill cancer cells by producing ROS and impairing the equilibrium of ion levels in cells. Importantly, metal-based nanoparticles have demonstrated the ability to neutralize acidity, generate oxygen, and reprogram pro-tumor M2 macrophages into antitumor M1 macrophages in the TME, which can transform the immunosuppressive TME into an immune-responsive milieu. These findings suggest that metal-based nanoparticles can potentially address the current efficacy issues of cancer immunotherapy with multipotent anticancer and immunomodulatory effects that are intrinsic to metal ions or generated by the on-demand design and fabrication of nanoparticles. Studies have also suggested that nanoparticle formulations are clearly beneficial in maximizing the potential of metalloimmunotherapy by improving the in vivo pharmacological profile of metal ions. The promise of metal-based nanoparticles can be fulfilled through the continuous development of metal ions and nanoparticle formulations to effectively regulate the TME in order to overcome the physical and immunobiological barriers and counteract immune evasion and the resistance of cancer cells.

The optimal formulation and clinical applications of metal-based nanoparticles will need to adapt to the design principles and general frameworks of nanomedicine. Precisely targeted delivery is a fundamental requirement of nanomedicine to increase the therapeutic window. Physiochemical properties such as composition, size, shape, surface charge, and functionality are key elements that regulate in vivo behavior and the targeted delivery of nanoparticles; thus, they must constitute the primary design parameters for the construction of novel nanomedicine formulations [89]. In addition, the on-target release of drug molecules and other functional nanoparticles in their active forms is essential for maximizing the drug-like properties of a nanomedicine. Furthermore, nanoparticles can be designed to decompose in response to specific signals such as high acidity, hypoxia, and enzymes that are exclusive or overexpressed in tumor tissues to allow for site-selective drug action that can decrease off-target toxicity and increase dose response [90]. The toxicity issue has been a critical hurdle for the clinical translation of nanomedicine; therefore, it must be considered in order to develop metal-based nanoparticles. The metal-based nanoparticles covered in this paper have been proven to be biocompatible when assessed according to body weight change, hematological marker levels, and/or histopathological analysis. Although preclinical studies have generally demonstrated the biocompatibility and safety of nanoformulations, these preclinical observations in mouse models often do not translate to human subjects because of the large physiological and anatomical gap, meaning that toxicity concerns are a major hindrance to the clinical application of nanomedicine. Nanoparticle surface modification is a common strategy to control specific and nonspecific interactions in vivo, thereby minimizing the off-target side effects and maximizing on-target therapeutic efficiency. Some approaches that have employed organic surface-coating layers or naturally derived cell membranes to modulate surface charge density, hydrophobicity, and functionality have been shown to ameliorate toxicity and improve the in vivo performance of nanoparticles [91]. Surface modification can also have a direct impact on nanoparticle activity [87]. Since many metal ions are essential nutritional elements that are opted into by the host, they are generally considered biocompatible; therefore, they can be less toxic drug candidates compared to other exogenous and synthetic agents [2]. Indeed, metal ions are well tolerated by animals; for example, the median lethal doses are 2000 mg/kg for Mn and Zn ions and 7500 mg/kg for Fe ion in mice, according to the information in the material safety data sheet provided by the manufacturers. However, nano-engineering modulates the physicochemical properties of metal ions, which are closely associated with their in vivo distribution, cellular responses, and toxicity profile. In addition, colloidal stability is another important factor that determines the in vivo behavior and toxicity of nanoparticles as the aggregation can alter their physicochemical properties. The preclinical examinations in the literature have generally reported that metal-based nanoparticles have good colloidal stability in a buffer and biological medium with hydrophilic surface modifications. Nonetheless, further investigations are required to test their long-term stability under practical transportation and storage conditions. Overall, how nanoparticle engineering affects the acute and chronic toxicity of metal ions remains to be thoroughly examined, and this must be investigated and optimized for individual nanoparticles with distinct physicochemical features. Accordingly, the toxicity issue will remain at the center of clinical applications of metal-based nanoparticles, and this issue should be carefully addressed along with the delivery issues. Currently, The United States Food and Drug Administration (FDA)-approved nanomedicines are mostly organic molecule-based nanoparticles composed of lipid and polymer. Although several iron-based nanoparticles have been approved by the FDA as inorganic and metal-based nanoparticles, they are indicated only for iron replacement therapy for the treatment of iron deficiency [92,93]. The FDA has published general guidelines for the production and clinical evaluation of new drug candidates; however, detailed regulations for the clinical development of novel inorganic nanoparticles, including metal-based nanoparticles, are yet to be established specifically for cancer immunotherapy applications.

The heterogeneity of cancer cells often makes a single intervention inefficient for controlling tumor growth. For more effective cancer treatment, the combination of multiple, non-overlapping treatments has been pursued to synergistically kill cancer cells through distinct cytotoxicity pathways. Metalloimmunotherapy can also play a role in combination therapy by harnessing several metal ions with unique immunostimulatory properties; for example, cGAS-STING activation (Mn, Zn), M1 macrophage polarization (Fe, Cu), and pyroptosis-induced immune responses (Ca, Na) can be simultaneously achieved by integrating the respective metal ions (mentioned directly above in parentheses) in a single nanoparticle formulation. Alternatively, metalloimmunotherapy can be applied in conjunction with other interventions that can enhance cancer cell elimination and anticancer immune responses via distinct therapeutic mechanisms, such as chemotherapy, RT, and other types of cancer immunotherapy. Some of the studies described in this review have already demonstrated the potential of combination therapy, reporting substantially improved therapeutic outcomes compared to individual treatments. For optimal combination therapy, combination agents and interventions should be rationally selected to maximize their synergistic efficacy with complementary anticancer activity. In addition, the novel engineering of nanoparticle formulations that support combination therapy would be of great interest and further facilitate the future development of metal-based nanoparticles.

## Data Availability

No new data were created or analyzed in this study. Data sharing is not applicable to this article.
